# Melatonin Regulates the Synthesis of Steroid Hormones on Male Reproduction: A Review

**DOI:** 10.3390/molecules23020447

**Published:** 2018-02-17

**Authors:** Kun Yu, Shou-Long Deng, Tie-Cheng Sun, Yuan-Yuan Li, Yi-Xun Liu

**Affiliations:** 1 State Key Laboratory of Stem Cell and Reproductive Biology, Institute of Zoology, Chinese Academy of Sciences, Beijing 100101, China; young137@163.com (K.Y.); dengsl@ioz.ac.cn (S.-L.D.); WuLiliwulw@163.com (T.-C.S.); WentingLi66@163.com (Y.-Y.L.); 2 National Key Laboratory of Agrobiotechnology, College of Biological Sciences, China Agricultural University, Beijing 100193, China

**Keywords:** melatonin, steroidogenesis, Leydig cell, Sertoli cell

## Abstract

Melatonin is a ubiquitous molecule and exhibits different effects in long-day and short-day breeding animals. Testosterone, the main resource of androgens in the testis, is produced by Leydig cells but regulated mainly by cytokine secreted by Sertoli cells. Melatonin acts as a local modulator of the endocrine activity in Leydig cells. In Sertoli cells, melatonin influences cellular proliferation and energy metabolism and, consequently, can regulate steroidogenesis. These suggest melatonin as a key player in the regulation of steroidogenesis. However, the melatonin-induced regulation of steroid hormones may differ among species, and the literature data indicate that melatonin has important effects on steroidogenesis and male reproduction.

## 1. Introduction

Spermatogenesis, the process of male gamete differentiation and maturation, is regulated by various hormones, such as luteinizing hormone (LH), follicle-stimulating hormone (FSH) from the pituitary gland, and testosterone produced by Leydig cells in the testis. FSH binds to its receptor restrictively expressed by Sertoli cells, which increases cAMP concentration and stimulates androgen-binding protein (ABP) synthesis and LH release, and this regulates testosterone production in Leydig cells. Testosterone concentration in the seminiferous tubules is associated with ABP [1]. Spermatogenesis is a mostly testosterone-dependent cellular event in the testis. In the absence of testosterone or androgen receptor, spermatogenesis fails to proceed beyond meiosis stage [2]. As rate-limiting steps, steroidogenic acute regulatory protein (StAR) and GATA binding factor 4 (GATA-4) co-regulate steroid hormone synthesis. Cyclic AMP response element binding protein (CREB) positively regulates the expression of steroidogenesis-linked genes [3]. Melatonin is a major secretory product of the pineal gland with both lipophilic and hydrophilic properties, and can pass through the blood–testis barrier and enter testicular cells. It acts through several specific receptors, including membrane melatonin receptors 1 (MT1) and 2 (MT2), and retinoid acid receptor-related orphan receptor Α (RORα), which has been identified in a large variety of mammalian cell types [4]. MT1 and MT2 couple with G-protein to regulate testosterone synthesis by regulating cAMP signal transduction cascades [5]. At present, there is still controversy about whether melatonin is directly combined with RORα to play its biological role [6]. However, melatonin can regulate animal reproduction at the transcriptional level through the nuclear receptor. Research suggests that aromatase could be activated by RORα and, as a result, conversion of androgen into estrogen was promoted [7].

The effects of melatonin on the levels of reproductive hormones are variable and depend on physiological conditions and species of animals [8]. During the long day period of seasonal breeders, such as rodents, melatonin reduces the expression of the androgen receptor and ABP [9]. Injecting melatonin into Syrian hamster testes during the breeding period significantly decreases the content of testosterone, decreases testicular volume, and diminishes androgen synthesis [10]. However, a constant supplementation of melatonin to the short-day breeding animals promoted the gonad function [11,12]. Long-term treatment with melatonin induces early testicular development in sika deer [13], and melatonin implants advance sperm production in silver fox [14]. Subcutaneous injections of melatonin increase testosterone concentrations in goats [15]. These processes eventually lead to spermatozoa maturation [16]. Melatonin is positively correlated with androgen concentration in the short-day period of the seasonal breeding animals. Our study revealed that, after treatment with melatonin, the concentration of testosterone in the somatic cells of sheep testes was 3.54 ± 0.17 nmol/L which was twofold higher than that in the control [17].

Reproduction is synchronized by day length via the pineal hormone melatonin. Melatonin affects stimulators of gonadotropin release hormone (GnRH) by regulating the secretion of hypothalamus–pituitary–gonadal related hormones, and then regulates the secretion of LH and FSH. Pinealectomy and exogenous melatonin for a long time on sheep could lead to the release of GnRH and LH, thus activating the reproductive activity [18]. For long-day mammals, melatonin is an inhibitory factor for reproduction, and there is evidence that the effect of this resistant gland is achieved by inhibiting GnRH [19]. At present, the exact mechanism of melatonin regulation of GnRH is still unclear [20]. Kisspeptins (Kp), a family of potent hypothalamic stimulators of GnRH neurons, is essential to convey melatonin’s message [21,22]. In short winter days, the Syrian hamster displays a complete gonadal atrophy with a marked reduction in expression of Kp. Acute peritoneal injection of Kp-induced c-Fos expression in a large number of GnRH neurons and pituitary gonadotrophs together with a strong increase in circulating testosterone [23,24]. In long days, Kp is highly expressed in the anteroventral periventricular nucleus (AVPV), with low expression in the arcuate nucleus in *Phodopus sungorus*. The situation is exactly the opposite in short days [25]. Photoperiod, via melatonin, modulates kiss1 signaling to drive the reproductive axis [26]. It is found that melatonin can induce the expression of Kiss1 and kiss2 and GnRH3 genes in zebrafish brain, and the increase of LHβ in the pituitary gland, which indicates that melatonin can promote gonad maturation and significantly improve the reproductive capacity of zebrafish [27]. Exogenous melatonin treatment for male sea bass found the expression of kiss1 gene was significantly increased in the hypothalamus after 30 days, the expression of kiss2 was enhanced after 90 days, but the expression of Kp in the back side of the brain was significantly decreased after 150 days, and decreased the mRNA expression of GnRH-1, GnRH-3, and FSH gene in the pituitary [28]. Melatonin can cause changes in the expression of Kp, thus affecting the changes in the reproductive system. The precise mechanisms which melatonin affects kisspeptin remain unclear. The path between melatonin and Kp is also a hot spot of current exploration [29].

## 2. Melatonin Regulates Leydig Cell–Testosterone Secretions

**Figure 1:**
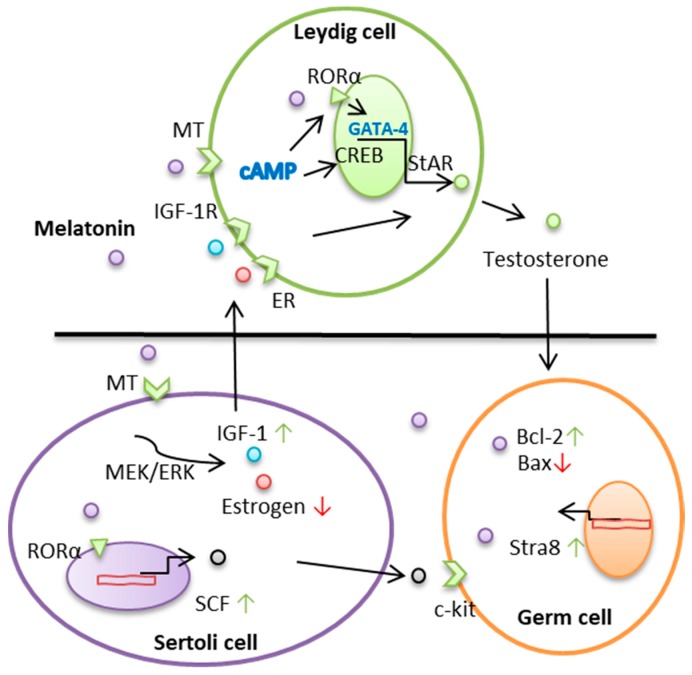
A schematic illustration of the melatonin regulates the synthesis of steroid hormones in rams.

Melatonin is involved in the function of the male reproductive system, particularly in the testes, since Leydig cells are sensitive to melatonin [30,31]. Melatonin regulates androgen secretion through a melatonin membrane receptor in Leydig cells [32]. The binding of phosphorylated CREB to the cAMP response element of the StAR promoter accelerates steroid synthesis. However, in steroidogenic cells, although not all cAMP-regulated genes, many of them have a regulatory sequence recognized by a GATA family transcription factor [33]. In the testes, GATA-4 predominantly regulates the transcription of StAR [34]. The inhibitory effects of melatonin on testosterone production are mediated by the downregulation of GATA-4 expression in a mouse Leydig cell line [10]. Our study shows that melatonin promoted testosterone production through RORα- enhanced GATA-4 expression in an in vitro goat spermatogonial stem cell differentiation culture system (Figure 1) [35]. Additionally, some studies have indicated that the increasing androgens may be primarily due to the stimulatory effect of melatonin on the steroidogenic enzyme 3β-hydroxysteroid dehydrogenase [36]. Other studies showed that activation of melatonin membrane receptors also increased the c-Jun-N-terminal kinase activity [37]. c-Fos and c-Jun were proposed to mediate the responses of Leydig cells to testosterone production in vivo [38]. These genes are involved in the StAR transcription [39].

## 3. Melatonin Regulates the Function of Sertoli Cells

The Leydig cell is a place where testosterone in the testis is synthesized and secreted, and regulated mainly by insulin-like growth factor secreted by Sertoli cells. Estrogen plays an important role in the function of testicular. Sertoli cells are the main source of estrogen production in immature males. Estrogen receptor-alpha (ERα) was expression in Leydig cells whereas ERβ was detected in Sertoli and germ cells, namely spermatocytes and spermatids [40]. The number of spermatogonial cells per testis was increased in ERβKO mice. The ERαKO mice had significant germ cell loss. The number of Leydig cells per testis was significantly increased in ERβKO mice but not in ERαKO mice [41]. The above results show that ERβ involved in regulation of Leydig cell proliferation and the production of testosterone in the adult mouse testis. The cytochrome P450 aromatase (P450arom) is a key enzyme responsible for the formation of estrogens from androgens and is exists in the endoplasmic reticulum of various tissues. P450arom has been immunolocalized in Leydig cells of numerous species as well as in germ cells of mouse, brown bear, and bank vole. Aromatase activity has been detected in vitro in immature and mature rat Leydig cells and Sertoli cells, while in pig, ram, and humans the enzyme activity is only present in Leydig cells. According to the stage of maturation of germ cell, the amount of aromatase transcripts decreases, being more elevated in younger than in mature rat germ cells [42]. Melatonin inhibits the activity and expression of aromatase, as well as decreases estrogen biosynthesis by regulating gene expression of aromatase via the promoter region [43,44,45]. We found that melatonin increased testosterone production in co-cultured Leydig and Sertoli cells from sheep. Melatonin increased the expression of stem cell factor and insulin-like growth factor-1 and decreased estrogen synthesis in Sertoli cells. It promoted insulin-like growth factor-1 and decreased estrogen content via the membrane melatonin receptor 1 [46]. Furthermore, melatonin regulates Sertoli cell metabolism and, thus, may affect spermatogenesis. Lactate produced by Sertoli cell provides nutritional support and has an anti-apoptotic effect in developing germ cells [47].

## 4. Conclusions

The melatonin receptor was expressed in testicular cells, and melatonin has effects on testicular development [48]. Serum and seminal plasma levels of melatonin are significantly lower in infertile patients [49]. In addition, environmental endocrine disruptors—such as estrogen analogues—significantly increased the rate of male infertility [50]. Male germ cells are extremely sensitive to reactive oxygen species (ROS), and excessive ROS can cause asthenozoospermia [51], however, melatonin can effectively reduce ROS and lipid peroxides. The simultaneous addition of melatonin during the transplantation of spermatogonial stem cells in azoospermia mouse testes increases the efficiency of transplantation and improves the structural properties of the testis tissue [52]. Further research on the mechanism of melatonin regulating the synthesis of steroid hormones and exploring the small molecules of melatonin receptors will help to cure the reproductive diseases caused by the disorder of steroid hormones.
